# Correlated duplications and losses in the evolution of palmitoylation writer and eraser families

**DOI:** 10.1186/s12862-017-0932-0

**Published:** 2017-03-20

**Authors:** Stijn Wittouck, Vera van Noort

**Affiliations:** 1 Centre of Microbial and Plant Genetics, KU Leuven, Leuven, Belgium; 2 Department of Bioscience Engineering, University of Antwerp, Antwerp, Belgium

**Keywords:** Post-translational modifications, Phylogenetic reconstruction, Tree reconciliation, Gene duplications, Gene losses, Correlated evolution

## Abstract

**Background:**

Protein post-translational modifications (PTMs) change protein properties. Each PTM type is associated with domain families that apply the modification (writers), remove the modification (erasers) and bind to the modified sites (readers) together called *toolkit domains*. The evolutionary origin and diversification remains largely understudied, except for tyrosine phosphorylation. Protein palmitoylation entails the addition of a palmitoyl fatty acid to a cysteine residue. This PTM functions as a membrane anchor and is involved in a range of cellular processes. One writer family and two erasers families are known for protein palmitoylation.

**Results:**

In this work we unravel the evolutionary history of these writer and eraser families. We constructed a high-quality profile hidden Markov model (HMM) of each family, searched for protein family members in fully sequenced genomes and subsequently constructed phylogenetic distributions of the families. We constructed Maximum Likelihood phylogenetic trees and using gene tree rearrangement and tree reconciliation inferred their evolutionary histories in terms of duplication and loss events. We identified lineages where the families expanded or contracted and found that the evolutionary histories of the families are correlated. The results show that the erasers were invented first, before the origin of the eukaryotes. The writers first arose in the eukaryotic ancestor. The writers and erasers show co-expansions in several eukaryotic ancestral lineages. These expansions often seem to be followed by contractions in some or all of the lineages further in evolution.

**Conclusions:**

A general pattern of correlated evolution appears between writer and eraser domains. These co-evolution patterns could be used in new methods for interaction prediction based on phylogenies.

**Electronic supplementary material:**

The online version of this article (doi:10.1186/s12862-017-0932-0) contains supplementary material, which is available to authorized users.

## Background

Protein palmitoylation is a PTM that involves the addition of a 16-carbon saturated fatty acid, called palmitate, to a cysteine residue in a protein [7]. Due to the discovery of the palmitoylation writer enzyme family in the early 2000s and the recent developments in the application of large-scale MS to study various PTMs, including palmitoylation, this PTM has only recently been studied intensively. The primary function of the cysteine-attached palmitoyl group is to serve as a lipid anchor on soluble proteins, turning them into peripheral membrane proteins. Different classes of lipids can function as lipid anchors [31], of which acyl groups and prenyl groups anchor proteins to the cytosolic side of a membrane. These acyl and prenyl groups often work together for stable and location-specific membrane attachment. Palmitoylation has a special position among them, as it is the only fully reversible lipid PTM, allowing for dynamics and regulation of protein-membrane interactions.

In addition to peripheral membrane proteins, integral membrane proteins are also frequently palmitoylated. Palmitoylated proteins are implicated in at least four classes of cellular processes. The first is the attachment of soluble proteins to the membrane and their localization to specific membrane compartments. The second function is the trafficking of membrane proteins between organelles and/or the plasma membrane (PM), and the third is the targeting of membrane proteins to specific parts of the PM such as postsynaptic clusters in neurons or lipid rafts. The fourth function is the stabilization of transmembrane proteins.

The principal protein family that is responsible for protein palmitoylation is the DHHC family of enzymes [18]. These are enzymes located in the membranes of the endomembrane system. They catalyze the transfer of a palmitoyl group from palmitoyl-CoA to a cysteine residue, forming a thioester bond. The first proteins of this family were discovered recently in yeast, and since then the family was found to be present in all eukaryotic species, with occurrences per genome ranging from less than ten in fungi to more than 20 in other eukaryotes [29]. The family is defined by its 51 residue DHHC domain, which is a variant of the C2H2 zinc finger motif. The DHHC domain containing proteins are characterized by i) a conserved sequence motif consisting of the residues aspartate, histidine, histidine and cysteine (DHHC) ii) six conserved cysteines iii) four to six transmembrane domains, the DHHC domain itself being located between two pairs of these on the cytosolic side of the membrane. This is compatible with the palmitate anchoring proteins to the intracellular side of the membrane in most cases.

Two small protein families are currently known to perform protein depalmitoylation: the acyl protein thioesterases (APTs) and protein palmitoyl thioesterases (PPTs) [7, 45]. In a structural/evolutionary classification of protein families, both are part of the alpha/beta hydrolase superfamily [32]. They make use of a nucleophile-acid-histidine catalytic triad. The APT and PPT families are sometimes situated within the serine hydrolase superfamily [38].

The APTs are cytosolic proteins [7]. Three of them have been found in humans: APT1, APTL1 and APT2. Originally they were identified as lysophospholipases (enzymes that deacylate monoacylated phospholipids), but recently they have all been found to perform depalmitoylation. APT1 is the best studied example [45]. It deacylates peripheral as well as integral membrane proteins, the classic example being signaling proteins of the Ras family. It shows some selectivity for substrates, and its efficiency varies across its substrates. It is strongly conserved among eukaryotes and, as opposed to proteins of the PPT and DHHC families, also present in prokaryotic species.

Enzymes of the PPT family have been shown to be targeted to the lysosomes [45]. They are conserved in eukaryotes. Humans have two of them: PPT1 and PPT2. They can both catalyze the depalmitoylation of palmitoyl-CoA, but only PPT1 is capable of depalmitoylating proteins. It has been shown that PPT2 is incapable of protein depalmitoylation [3]. PPT1 has been heavily studied because it has been identified as the causative gene of the disease infantile neuronal ceroid lipofuscinosis [24]. Neuronal ceroid lipofuscinoses (NCL) are a set of diseases characterized by an aggregation of so-called lipofuscin granules in neurons. These lipofuscin granules are composed of residues from lysosomal digestion. The nature of infantile NCL as lysosomal storage disorder seems very compatible with the function of PPT1 as a lysosomal enzyme. In non-diseased individuals, the protein is glycosylated at three places and transported to the lysosomes via the mannose 6-phosphate (M6P) pathway [24]. Nevertheless, many studies have shown that PPT1 is also implicated in processes outside of the lysosome. In neurons, the enzyme has sometimes been found in synaptic vesicles. It is often secreted (also in non-neuronal cells) and endocytosed again via the M6P receptor. However, much of the function of the protein remains to be discovered.

A lot less is known about protein depalmitoylation than is known about palmitoylation; it is very likely that other depalmitoylating enzymes remain to be discovered [7].

Explaining the seemingly irreducible complexity of writer-eraser-reader systems is an important challenge that needs to be addressed for every PTM type under investigation. In the case of tyrosine phosphorylation, it has been speculated that primitive PTPs in species without PTKs or reader domains are active to dephosphorylate Tyr residues that have been *accidentally* phosphorylated by promiscuous Ser/Thr kinases [30]. A prototypical example of this phenomenon is seen in *Saccharomyces cerevisiae*, where accidentally occurring pTyr residues exert unwanted allosteric effects, causing selective pressure for their removal [28].

Similarly to tyrosine phosphorylation, the very first form of lysine acetylation was also probably accidental. Acetylation has been shown to occur non-enzymatically in vitro and probably also in vivo [44]. In *E. coli*, acetylation levels reflect levels of acetyl-CoA present in the cell. This cofactor reflects, in turn, the nutrient status of the cell due to its central position in cellular metabolism. Deacetylation is performed by sirtuins, which allows for further regulation (i.e. in response to other signals than acetyl-CoA levels). The earliest acetylation system in the last universal common ancestor could have been similar to this system in *E. coli*. In this evolutionary scenario, the acetylation writer and reader domains have been later additions to this primitive but already functional PTM system using only eraser enzymes.

While for some PTM types like tyrosine phosphorylation and lysine acetylation there have been speculations on their stepwise origin, this is not the case for most of the PTM types, including palmitoylation. In this work we study the origin and evolution of the palmitoylation toolkit enzymes. The main aims are to identify lineage-specific family expansions and contractions and relate those to protein palmitoylation functionality and secondly to identify if the evolutionary histories of palmitoylation toolkit enzymes are correlated. If more studies like this accumulate it will become clear if the evolution of PTM systems on the long timescale follows general trends, such starting out as an accidental modification that gives rise to erasers as first of the toolkit domains.

## Results

### Profiles of (de-)palmitoylating protein families

**Fig. 1:**
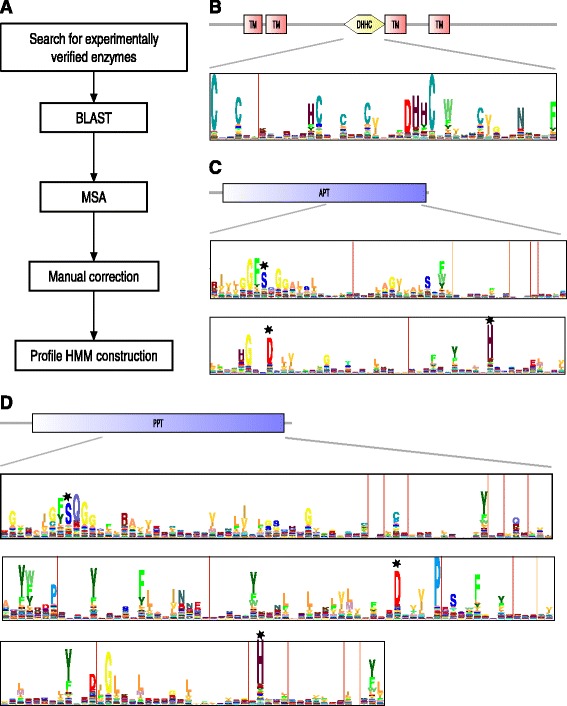
Profiles of palmitoylating and depalmitoylating enzymes. **a** Overview of the strategy for Profile HMM construction. **b** Domain composition and HMM profile of DHHC protein domain. **c** Domain composition and HMM Profile of APT protein family. **d** Domain composition and HMM profile of PPT protein family

We first searched the literature for proteins for which there is experimental evidence for palmitoylating or depalmitoylating enzymatic activity (Fig. 1a). We found five members in three species of the APT family with depalmitoylating activity (Table 1), four members in four species of the PPT family with depalmitoylating activity and 30 members in two species of the DHHC family with palmitoylating activity. The APT1 of *Saccharomyces cerevisiae* and *Toxoplasma gondii* are homologous to human APT1. For one of the palmitoylating enzymes ZDHHC13 the activity is still doubtful; the protein has been shown to be autopalmitoylated but there is no direct evidence for its palmitoylating activity. There is one more human copy of the DHHC family ZDHHC11B but its activity has not been studied.

**Table 1:** Palmitoylation writer and eraser proteins of the APT, PPT and DHHC families of (de-)palmitoylating enzymes whose activity has been experimentally demonstrated

Family	species	gene	Reference
APT	*Saccharomyces cerevisiae*	APT1	[13]
	*Homo sapiens*	APT1	[11]
		APTL1	[41]
		APT2	[42]
	*Toxoplasma gondii*	APT1	[21]
PPT	*Bos taurus*	PPT1	[5]
	*Homo sapiens*	PPT1	[9]
	*Drosophila melanogaster*	Ppt1	[17]
	*Caenorhabditis elegans*	ppt-1	[35]
DHHC	*Saccharomyces cerevisiae*	AKR1	[23]
		ERF2	[1]
		SWF1	[43]
		PFA3	[39]
		PFA4	[26]
		PFA5	[19]
	*Homo sapiens*	ZDHHC1	[41]
		ZDHHC2	
		ZDHHC3	
		ZDHHC4	
		ZDHHC5	
		ZDHHC6	
		ZDHHC7	
		ZDHHC8	
		ZDHHC9	
		ZDHHC11	
		ZDHHC12	
		ZDHHC13	
		ZDHHC14	
		ZDHHC15	
		ZDHHC16	
		ZDHHC17	
		ZDHHC18	
		ZDHHC19	
		ZDHHC20	
		ZDHHC21	
		ZDHHC22	
		ZDHHC23	
		ZDHHC24	

BLAST searches in proteomes of completely sequenced organisms resulted in 1576 DHHC sequences, 144 APT sequences and 136 PPT sequences. Based on a subset of these homologs, we generated multiple sequence alignments and manually corrected those. These seed alignments contained 159 DHHC sequences, 134 APT sequences and 128 PPT sequences. We used the corrected alignments to construct Hidden Markov Models (HMMs) for each of the three families (Fig. 1b–d). The DHHC residues as well as six conserved cysteines can clearly be observed in the profile of the DHHC family (Fig. 1b) that has 43 match states. Other conserved residues are a histidine at position 13, an aromatic residue at position 30, asparagine at 39 and phenylalanine at 43. The APT and PPT profiles are much longer: 207 and 246 match states respectively (Fig. 1c–d). The actual domains are even slightly longer, because there are some insertion states that span multiple positions. Apart from the catalytic triads, the two profiles show other common characteristics. For example, the catalytic serine is located in an area of conserved hydrophobic residues. They also both contain many sites with conserved aromatic residues (green residues in the logos), especially the PPT profile that has more than 10 of these.

The length of the DHHC family being short is consistent with the multi-domain nature of these proteins (Fig. 1b) as opposed to the single domains of which the APT and PPT family consist. Palmitoyltransferases contain next to the DHHC domain, multiple Transmembrane domains that anchor them to the membrane.

### Phylogenetic distribution

We used the HMM-profiles to search for protein family members in completely sequenced genomes of 119 eukaryotic and 1008 prokaryotic species. A length-score distribution was used to determine a threshold for inclusion and proteins not containing essential residues were removed. We found that metazoan genomes contain a large number of DHHC encoding genes, although there is considerable variation between species. Most of them have 10 to 25 DHHC genes, whereas vertebrate genomes contain at least 15 DHHCs. All eukaryotic species contain between 1 and 4 APTs (with one exception of zero) and zero to four PPTS (with one exception of six). Fungal genomes contain the smallest number of DHHCs; only three to seven.

**Fig. 2:**
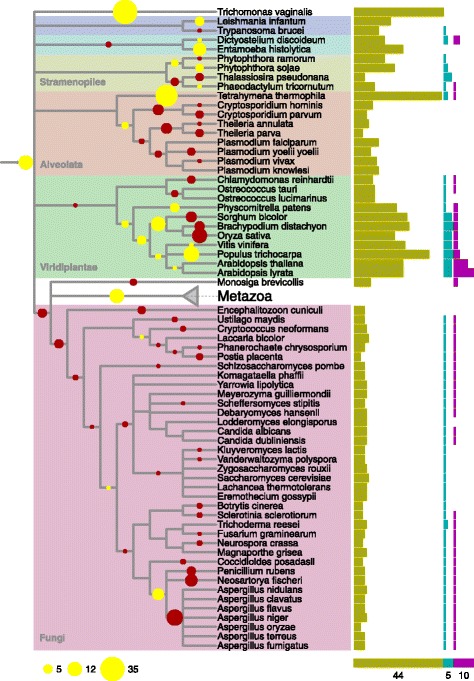
Evolutionary history of DHHC family proteins. Tree reconciliation of the DHHC rearranged tree. *Green bars* indicate number of DHHC protein in the extant genome, cyan APT proteins, and magenta PPT proteins. *Yellow circles* indicate inferred increases in copy numbers of the DHHC family, *red circles* indicate inferred decreases in copy numbers of the DHHC family. The tree topology is extracted from NCBI taxonomy. Tree continues in Fig. 3

Within the green plants (Viridiplantae) there is a clear distinction between two classes; the Chloryphyta have few DHHCs, one APT and one PPT whereas the Embryophyta (land plants) have many DHHCs, four or five APTs and two or three PPTs (Fig. 2). Only the APT family was found outside of eukaryotes with some proteobacteria having one or two copies of this family.

**Fig. 3:**
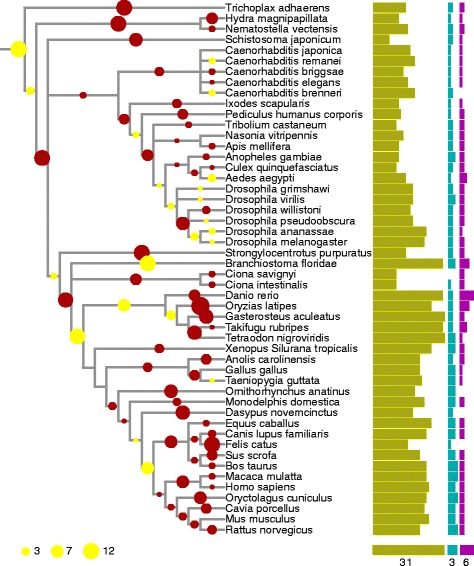
Evolutionary history of DHHC family proteins. Tree reconciliation of the DHHC rearranged tree. *Green bars* indicate number of DHHC protein in the extant genome, cyan APT proteins, and magenta PPT proteins. *Yellow circles* indicate inferred increases in copy numbers of the DHHC family, *red circles* indicate inferred decreases in copy numbers of the DHHC family. The tree topology is extracted from NCBI taxonomy. Tree continued from Fig. 2

From the phylogenetic distribution (Figs. 2 and 3) it is already clear that the number of copies in a genome of each of the enzyme families is correlated, the correlation being 0.58 between DHHC and APT; 0.43 between DHHC and PPT and 0.53 between APT and PPT. However, such correlation could be due to few phylogenetic events; the distributions of DHHC, APT and PPT genes in extant genomes are not phylogenetically independent.

### Duplications and losses in gene family trees

We used phylogenetic reconstructions and tree reconciliation to identify phylogenetically independent duplication and loss events and mapped these to a species tree. Bias in inferred duplication and losses can be introduced by small errors in gene trees. To reduce this bias, we performed gene tree rearrangement such that branches that are uncertain are rearranged in order to follow the species tree (see methods). If there are many duplications in a specific branch, the gene family is expanding whereas many losses in one branch result in contraction of the family. We identify an expansion of DHHC at the last eukaryotic common ancestor. This means part of the diversity in present day DHHC enzymes arose already in this earliest stage of eukaryotic evolution. At the root of the metazoan, the DHHC diversity was shaped by an early expansion followed by contractions. These contractions continued in the non-chordate eukaryotic species and led to their low DHHC numbers. Some of these species have a slightly larger number of DHHCs due to small late expansions. In the Chordata, the early eukaryotic contractions were followed by expansions. One expansion is visible in the lancelet lineage, leading to the species *Branchiostoma oridae* . A second one is actually a stretch of expansions, starting at the Euteleostomi (Vertebrata) and continuing in two lineages: via the Clupeocephala until the Percomorphaceae ancestor and via the Tetrapoda until the Boreoeutheria ancestor. This stretch of expansions was followed by late losses in all lineages. This leads to the conclusion that the copy number of DHHCs peaked in at least three ancestral species. First in the common eukaryotic ancestor, that appears to have had a larger number of DHHCs than the single cell eukaryotes and Ecdysozoa living today. After that in the common ancestors of the Clupeocephala and Boreoeutheria, that both had larger DHHC counts than any eukaryotic species sequenced today. In the superphylum of the Alveolata, small contractions in the apicomplexan linages led to a small number of DHHCs.

In the APT and PPT families we also observe gains and losses but the copy number per genome is much smaller than the DHHC (Additional files 1 and 2: Figures S1 and S2). In the APT family we observe expansions at the last common eukaryotic ancestor, at the metazoan ancestor and the ancestor of the euteleostomi. The apicomplexa lost all APT family members, likely linked to their parasitic lifestyle.

### Correlation between DHHC and APT/PPT evolution

**Fig. 4:**
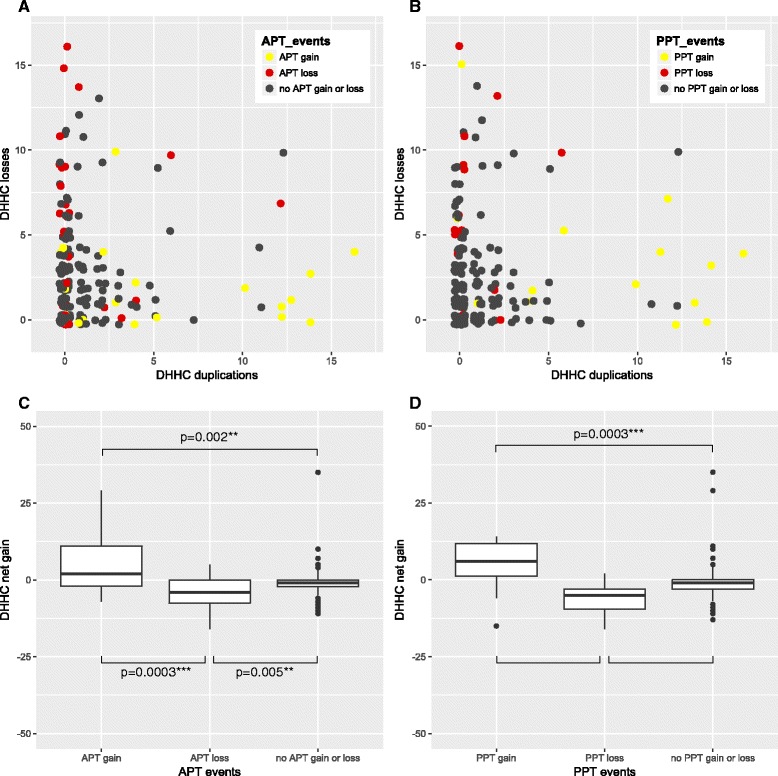
Correlated evolution of DHHC, APT and PPT families. **a**, **b** Each dot represents one branch in the species tree. X-axis number of duplications in the DHHC family in that branch, y-axis number of DHHC losses in that branch (**a**) *yellow*; branch with gains in APT family, *red*; branch with losses in APT family, *black* no change in APT family. **b** *yellow*; branch with gains in PPT family, *red*; branch with losses in PPT family, *black* no change in PPT family. **c** Significant differences in net gain in DHHC family per branch between three categories of branches, gain in APT, loss in APT or no change in APT family. **d** Significant differences in net gain in DHHC family per branch between three categories of branches, gain in PPT, loss in PPT or no change in PPT family

The phylogenetic placing of evolutionary events of the DHHC, APT and PPT families are strikingly similar. At species tree branches with many DHHC duplication events and few losses, the APT family also often expanded (Fig. 4a). Conversely, at branches with few DHHC duplications and a lot of losses, the APT family often reduced (Fig. 4a). The association between the two families is only apparent for species tree branches with a large number of duplications or losses; the pattern is not visible for species tree branches with less than five DHHC duplications and less than five losses.

We assessed statistical significance by considering the net expansions in the DHHC family for each internal branch and dividing them into three groups according to events in APT or PPT family; expansion, contraction or no change. We used the Wilcoxon rank-sum test for independent samples. The association between evolutionary events in the DHHC family with events in the APT family is significant (Fig. 4c, *p* = 0.0011). The association between DHHC and PPT evolutionary events (Fig. 4b) is even stronger (Fig. 4d, *p* = 0.0001). In addition, the association between the APT and PPT events is also significant (Fisher exact test; *p* = 2e-7).

For comparison, reconciliation of the ﻿original ML DHHC tree without rearrangement can be found in Additional file 3: Figure S3. As a negative control, we also inferred the evolutionary history of the histone deacetylase (HDAC) enzyme family that has 710 members in the genomes we analyzed. We tested its association with the APT and PPT families in the same manner as we did for the DHHC family. Although some level of association is observed, there is no significant correlation between HDAC net gain and changes in the APT and PPT families (Additional file 4: Figure S4).

## Discussion

In this work we have analyzed the phylogenetic distribution of palmitoylating and depalmitoylating enzymes and show that these families co-evolve. We find that the DHHC family is only present in eukaryotes. The APT family on the other hand is also present in bacteria. More specifically, we find two clusters of proteins matching the APT model: a high scoring cluster with exclusively proteins from the Proteobacteria, and a lower scoring cluster with proteins from Proteobacteria as well as other bacterial clades. The presence of APTs in bacteria strongly suggests that they arose before the DHHCs in the course of evolution. What could the function of this palmitoylation eraser family be without there being palmitoylation writer enzymes? A possibility is that they have evolved to remove accidental palmitoylation, as this modification has been shown to occur non-enzymatically. This removal of accidental modifications is similar to what has been speculated for tyrosine phosphorylation and lysine acetylation erasers [2].

In the DHHC family, we find consecutive periods of expansions and contractions. A first hypothesis to explain the expansion-contraction patterns is a temporary selection pressure for a larger number of DHHCs. In general, the adaptive expansion of a gene family can occur for two reasons: a dosage increase of the proteins or a functional diversification of the family [12]. Contractions of gene families are thought to be mainly the result of neutral selection; in other words, the loss alleles are fixated by random drift because they are not deleterious.

In this light, the question becomes why contractions of the DHHC family after its expansion are not disadvantageous. Actually, Hogeweg and co-worker have shown that in an evolving system, lineages with whole genome duplication are better able to adapt to a changing environment [10]. In case of expansion for dosage increase, it could be speculated that gene expansion is the fastest way to achieve this dosage increase. Amplification of an, initially, low-efficiency enzyme can result in adaptive mutations to arise in the enzymatic function or the regulation in any of the gene duplicates. As the occurrence of adaptive mutations is limited by the per base mutation rate, a duplication increases the options to adapt. Over time, the expression levels or the enzymatic efficiency of the individual genes is optimized to the new function, rendering the extra copies superfluous. In case of functional diversification, the explanation for gene loss might be that after new and improved types of DHHCs have evolved from duplicated genes, they partly replace the functions of the conserved types, also rendering them obsolete.

The co-occurrence of DHHC and APT expansions fits in this selection hypothesis in two possible ways. First, the APT expansions likely comprised only one or two duplications. The evolution of a new palmitoylation eraser enzyme might have created opportunities for more extensive use of palmitoylation in general, leading to selection for more DHHCs. Alternatively, another molecular invention or a change in some environmental factor might have created a selection pressure for the palmitoylation machinary in general, including both writers and erasers.

In contrast to the APT family, the strong co-evolution of the PPT family with the DHHC and APT families was rather unexpected, for a number of reasons. Firstly, it is fairly certain that PPTs reside in the lysosomes. Therefore, they are unlikely to participate in any signaling processes and it is unclear why their diversification in function might be useful in case of increased use of protein palmitoylation, although selection pressure for duplications for dosage increase is conceivable. And secondly, very few PPT enzymes are experimentally confirmed as palmitoylation erasers, and for some of them it has even been shown that they are not capable of this function, such as human PPT2. If the selection scenario is correct, this might be an indication that protein depalmitoylation is in fact the main function of the PPT family, and that these non-depalmitoylating PPTs are rather the exception.

A pattern of expansion followed by contractions is often seen at whole-genome duplications (WGDs). A possible pitfall of this analysis is that we simply observe the effect of WGDs. In Metazoa, known WGDs occurred in the common ancestor of the Vertebrata and in the common ancestor of the teleost fishes [34]. Also in the history of land plant evolution, one or more WGDs are known to have occurred at the origin of multiple clades or species that are present in our data: the Poaceae, eudicots, *Arabidopsis thaliana*, *Populus trichocarpa* and *Physcomitrella patens* [34]. Although some overlap is visible with DHHC expansions and WGD events, DHHC expansions also occurred when no WGD took place and gene family expansions continue in clades after WGD events.

## Conclusions

This study and previous studies suggests that the functional link between writer and eraser domains is reflected in correlated evolution on the level of duplications and losses. Conversely, this information could be used to infer functional relationships. So far, prediction of functional interaction based on phylogenetic information has been based on correlated sequence evolution or correlated presence-absence profiles (for a review see [22]) but not on duplications and losses. These methods work well specifically for bacteria and archaea but not as well in eukaryotes [15]. The co-evolution patterns we find here, could be employed to further develop functional interaction prediction methods specific to eukaryotes.

## Methods

### Experimentally validated enzymes

The literature resources Scopus and KU Leuven LIMO were searched for research articles describing DHHCs, APTs and PPTs using the search terms protein acylation; protein depamitoylation; protein acyl thioesterase and palmitoyl protein thioesterase . We read the articles and stored protein and species names of characterized enzymes in a table.

### Protein sequences

We downloaded all protein sequences from the STRING database (version 9.1) [16]. Locally installed BLAST (version 2.2.30) [4] was used to find homologs of the experimentally validated APT and PPT proteins, with an *e*-value of 10^−50^. To find homologs of the DHHC family an *e*-value cut-off of 10^−10^ was used. Other search paramaters were default; gap opening penalty 11, gap extension penalty 1, word size 6 and the BLOSUM62 substitution matrix. For the APT and PPT families, the hydrolase domains make up the largest part of the proteins. Thus, whole protein sequences were used as queries. For the DHHC family only the DHHC domain was used as query. Non-redundant BLAST results were collected together for each set of experimentally confirmed enzymes with the same function.

### Construction of seed alignments

The MAFFT package version 7 [20] contains three algorithms: the FFT-NS-i, L-INS-i and G-INS-i. All of these are based on a progressive alignment using a guide tree, followed by iterative refinement. FFT-NS-i is the fastest of the three methods. L-INS-i and G-INS-i are slower but more accurate.

The BLAST search results of the DHHC family resulted in a large number of protein sequences.

Not all of these sequences are needed to capture the common characteristics and the diversity of the family. Taking a subset has the advantage of being able to create a more accurate alignment, more atypical sequences are excluded and it is easier to remove problematic sequences.

We implemented a subset function in R and makes use of the R package Phangorn [37]. The function starts with reading the MSA and the construction of a distance matrix, making use of maximum likelihood distance estimation and the LG model of amino acid substitution. Then follows an iterative process of two steps. First, the two sequences with the smallest distance between them are identified. And then, of these two, the sequence with the largest total distance to all other sequences is removed. These two steps are repeated until the number of sequences is reduced to the required number. For the DHHC family, the BLAST search results were first aligned with the fast MAFFT FFT-NS-I method. Then, a subset of 200 sequences was extracted using the subset method.

The sequences in this subset were then aligned with the accurate L-INS-i algorithm. Next, the alignment was inspected and doubtful sequences were removed. The criteria for inclusion were: the presence of multiple cysteine residues in the DHHC domain, the DHHC motif itself and the 2x2 transmembrane structure of the proteins. For the prediction of the transmembrane structures, we used the TMHMM server, version 2.0 [25].

For the alignments of the APT and PPT families, the G-INS-i algorithm was used. While for the DHHC family, L-INS-i seemed to give better results than G-INS-i, the opposite was true for the PPT and APT families. The reason for this is that for these families, a much larger portion of the sequences was alignable, and thus it is appropriate to include global instead of local pairwise alignment information in the iterative optimization process. We made accurate alignments for the APT and PPT families by making use of the multithreading option implemented in MAFFT. First, we aligned the full set of BLAST results with an extended G-INS-i algorithm, using 10,000 optimization cycles. Then we manually inspected the resulting MSA. We made small corrections to the alignment and excluded some sequences based on the knowledge that the catalytic triad is an essential feature of the protein family.

### Profile HMMs

For the construction of profile HMMs and the subsequent database searches using these profiles, we used the HMMER software, version 3.1b1 [14]. By default, the hmmbuild command will select all columns of the alignment that contain less than 50% gaps and model these as the match states. To increase the specificity of the search, columns with many gaps or low conservation were excluded. A strict non-gap percentage threshold of 80% and a conservation threshold of 5*10^−6^ were applied. These selection criteria were implemented by the trimAl software, version 1.2rev59 [6]. The boundaries of the family domain were determined by visual inspection of the multiple sequence alignments and columns outside of these boundaries were excluded. Skylign was used to visualize the profile HMMs.

### HMMER searches

The constructed profile HMMs were used to search the protein sequence database. The domain list output of hmmsearch was used for further analysis. Inclusion thresholds were set based on i) visual inspection of the length-score plot, ii) sequence characteristics of the results iii) functional annotations. After setting the inclusion thresholds domain hits were removed that did not contain essential parts of the domain, the DHHC motif or the catalytic triad.

### Phylogenetic reconstruction

To construct a multiple alignment of the DHHC family, first complete domain hits without large insertions or deletions were aligned with each other with the accurate L-INS-i algorithm of MAFFT. Other sequences were added with the –add option. Prealigned L-INS-i DHXC sequences were added with the –addprofile option. Alignment columns with less than 0.5% residues (99.5% gaps) were removed to save computation time. The APT and PPT sequences were aligned in one step with the MAFFT G-INS-i algorithm with 10,000 optimization cycles.

For the construction of all phylogenetic trees, we used version 8.1.21 of RAxML [40]. The Pthreads implementation was used for parameter optimization, while we used the hybrid implementation for the actual tree inferences. We determined the optimal protein substitution model using a script provided by A. Stamatakis on the RAxML website. The script determines the substitution model that results in the highest likelihood value of a fixed maximum parsimony (MP) tree. The initial rearrangement parameter determines the depth of the tree search in each iteration of the search algorithm. The RAxML software contains an option to determine this parameter automatically, but the manual advises to test this automatic option versus a fixed setting of 10 for a couple of trees. The option (fixed or automatic) that results in the tree with highest likelihood value should be used for the final tree inferences. We generated five MP starting trees and tested both the automatic and fixed options on each of the trees. A fixed rearrangement setting of 10 gave the best results for all families.

For the construction of phylogenetic trees for fewer than 1000 sequences, the RAxML manual advises the use of the analyses invoked by the -f a option, which combines rapid bootstrapping with an extensive search for the ML tree. We used this strategy for the APT and PPT families. The algorithm starts by computing bootstrap trees with RBS enabled. It then uses these bootstrap trees as starting points to explore the tree space in three steps, increasing the depth of the search but decreasing the number of trees in each step. First, it does a fast search, using every fifth bootstrap tree as a starting tree. In the second step, the ten most promising results of the fast searches are further improved by doing slower optimizations. Finally, the best of these resulting trees is thoroughly optimized. This last step always uses the gamma model of rate heterogeneity, even when the CAT option is specified. The approach above is less attractive for large trees. One reason for this is that it takes a lot of computation time, and another that the required time is impossible to predict. Therefore we used a slightly different strategy for the DHHC family. We started by computing bootstrap trees in batches of 100 (with RBS enabled). After each batch, we combined the bootstraps of all batches and tested the MRE criterion. We stopped when the criterion converged.

To find the ML tree, we did 20 searches starting from independent parsimony starting trees. To estimate the irregularity of the likelihood surface, we calculated the average Robinson-Foulds distance (RF) as well as the average WRF between all trees. This resulted in a RF of 10.9% and a WRF of 2.8%. These values indicate that while the trees differ quite substantially in general, they are very similar at their highly supported branches. For this reason, we did not perform extra ML tree searches. To obtain the final tree, we picked the ML tree with the highest likelihood between these 20 trees and the trees obtained in the process of tuning the rearrangement setting. We then used the -f b option to draw the bootstrap confidence values on this tree.

RAxML tree construction algorithms always produce unrooted trees. We used a rooting algorithm built into RAxML. It uses a variant of midpoint rooting; the tree is rooted in such a way that the sums of the branches of both subtrees of the root are equal.

### Tree rearrangement, reconciliation and mapping

The first step was the preprocessing of the ML gene tree using R. We rooted the tree with bootstrap values on the root given by the RAxML rooted tree. We gave names to the internal nodes of the tree to make later tracking easier and uniform. Tree rearrangement was carried out with NOTUNG [8]. NOTUNG is a gene tree-species reconciliation software package that supports duplication-loss event models with a parsimony-based optimization criterion. It thus identifies the smallest (weighted) number of independent evolutionary events that explain the phylogenetic gene tree. NOTUNG functions include rearranging of a rooted gene tree in areas of weak sequence support, thus avoiding overestimating duplications in gene trees that are incongruent with the species-tree. We used the standard parsimony weight parameters of the software: 1.5 for a duplication, 0.0 for a conditional duplication and 1.0 for a loss. For the bootstrap cut-off value to identify weak branches, the value of 90 was used. This is a relatively strict value. For the rearrangement procedure, a binary species tree is needed. We obtained binary species trees for each gene family by extracting the NCBI taxonomy species identifiers of all species present in the gene tree and uploading them to the phyloT online tree generator (biobyte solutions GmbH, 2014) to generate a binary species tree [36]. We used the following options in phyloT: NCBI taxonomy IDs as identifiers, collapsed internal nodes, no polytomies (this option randomly resolves the polytomies of the underlying non-binary species tree from NCBI), newick format.

After the tree rearrangements we performed the reconciliation, both on the raw ML gene tree and on the rearranged gene tree. For this step we needed non-binary species trees; these were generated using phyloT with the same options as described in the previous paragraph, except that the polytomies were retained. For the inferred duplications, NOTUNG outputs a lower and an upper bound. The lower bound represents the oldest species in which the duplication was present; the upper bound is the youngest species in which the duplication was not present. The losses are written to a table with the species node names and the number of losses.

The fact that the gene trees were rearranged using a species tree with randomly resolved polytomies, means that some random rearrangements were introduced in the gene tree. This is however no problem, because in the reconciliation process the non-binary species tree was used. The random rearrangements then corresponded again to polytomies in the species tree, meaning that they can only have led to conditional duplication inferences. These were not retained in the results.

The inferred duplication and loss events were mapped on a species tree and visualized with the online tool iTol [27]. The conversion of the event data to the suitable format for iTol was done in R. We used the DHHC species tree for data visualization since it contained all eukaryotic species in the database that we used. In both the event data and the species tree, the NCBI taxonomy identifiers of the species were converted to the species names. To handle phylogenetic trees in R, we made use of the APE package [33].

### Evolutionary history of the HDAC family

An HMM of the histone deacetylase domain was retrieved from Pfam (PF00850). The HMMER search, construction of phylogenetic tree, rearrangement and reconciliation steps were performed in the same way as for the APT and PPT families. In total 710 HDAC sequences were included in the phylogenetic tree. We set a fixed value of 200 bootstraps in the tree inference process.

## Additional files

Additional file 1: Figure S1. Tree reconciliation of the APT ML and rearranged trees. The upper half of the circles represents the results using the ML tree; the lower half represents the results from the rearranged tree. Yellow semicircles indicate inferred gain in the APT family, red semicircles indicate inferred losses in the APT family. Black indicates no inferred copy-number changes. The tree topology is extracted from NCBI taxonomy. (PDF 41 kb)
Additional file 2: Figure S2. Tree reconciliation of the PPT ML and rearranged trees. The upper half of the circles represents the results using the ML tree; the lower half represents the results from the rearranged tree. Yellow semicircles indicate inferred gains of the PPT family, red semicircles indicate inferred gene losses of the PPT family. Black indicates no inferred copy-number changes. The tree topology is extracted from NCBI taxonomy. (PDF 38 kb)
Additional file 3: Figure S3. Tree reconciliation of the DHHC ML tree (without rearrangement). Yellow circles indicate inferred increases in copy numbers of the DHHC family, red circles indicate inferred decreases in copy numbers of the DHHC family. The tree topology is extracted from NCBI taxonomy. (PDF 49 kb)
Additional file 4: Figure S4. Histone deacetylase family as a negative control. A) Differences in net gain in HDAC family per branch between three categories of branches, gain in APT, loss in APT or no change in APT family B) Differences in net gain in HDAC family per branch between three categories of branches, gain in PPT, loss in PPT or no change in PPT family (PDF 41 kb)
