# Supplementary figures and images for: Correlated duplications and losses in the evolution of palmitoylation writer and eraser families
Source: BMC Evol Biol. 2017 Mar 20;17:83. doi: 10.1186/s12862-017-0932-0 (PMC5359973; doi:10.1186/s12862-017-0932-0)

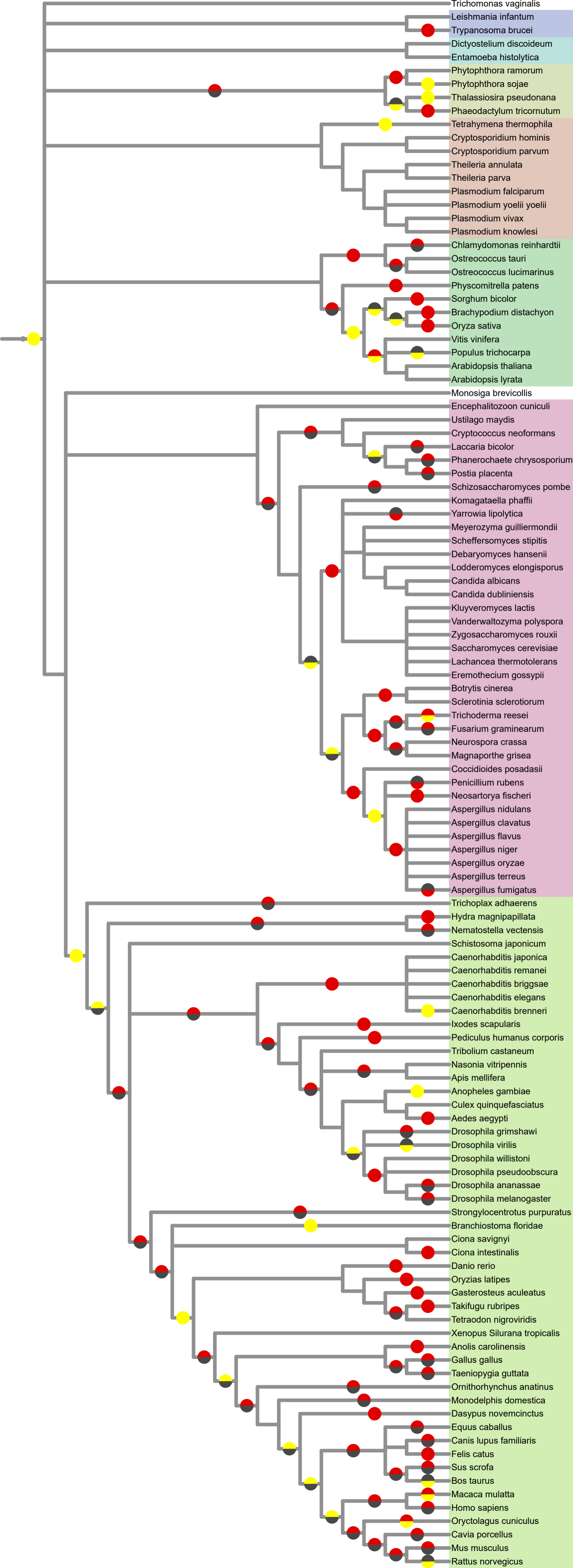

Supplement: Additional file 1: Figure S1. — Tree reconciliation of the APT ML and rearranged trees. The upper half of the circles represents the results using the ML tree; the lower half represents the results from the rearranged tree. Yellow semicircles indicate inferred gain in the APT family, red semicircles indicate inferred losses in the APT family. Black indicates no inferred copy-number changes. The tree topology is extracted from NCBI taxonomy. (PDF 41 kb) [file 12862_2017_932_MOESM1_ESM.pdf]

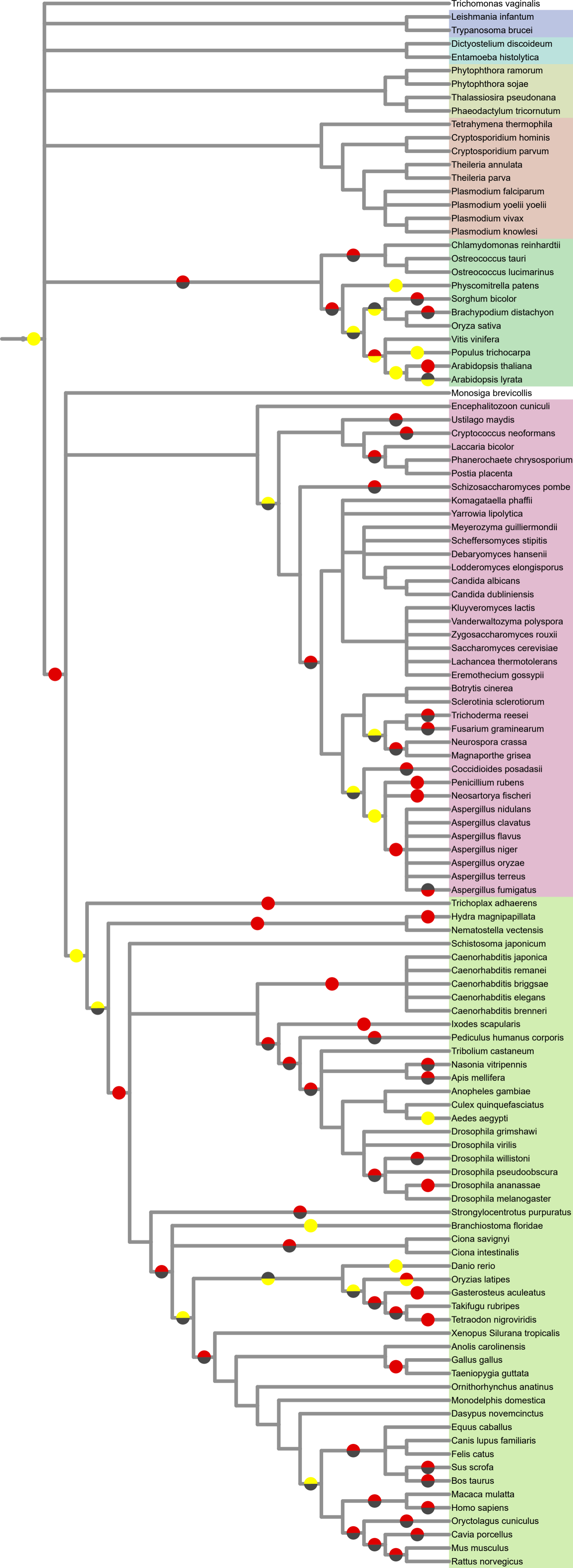

Supplement: Additional file 2: Figure S2. — Tree reconciliation of the PPT ML and rearranged trees. The upper half of the circles represents the results using the ML tree; the lower half represents the results from the rearranged tree. Yellow semicircles indicate inferred gains of the PPT family, red semicircles indicate inferred gene losses of the PPT family. Black indicates no inferred copy-number changes. The tree topology is extracted from NCBI taxonomy. (PDF 38 kb) [file 12862_2017_932_MOESM2_ESM.pdf]

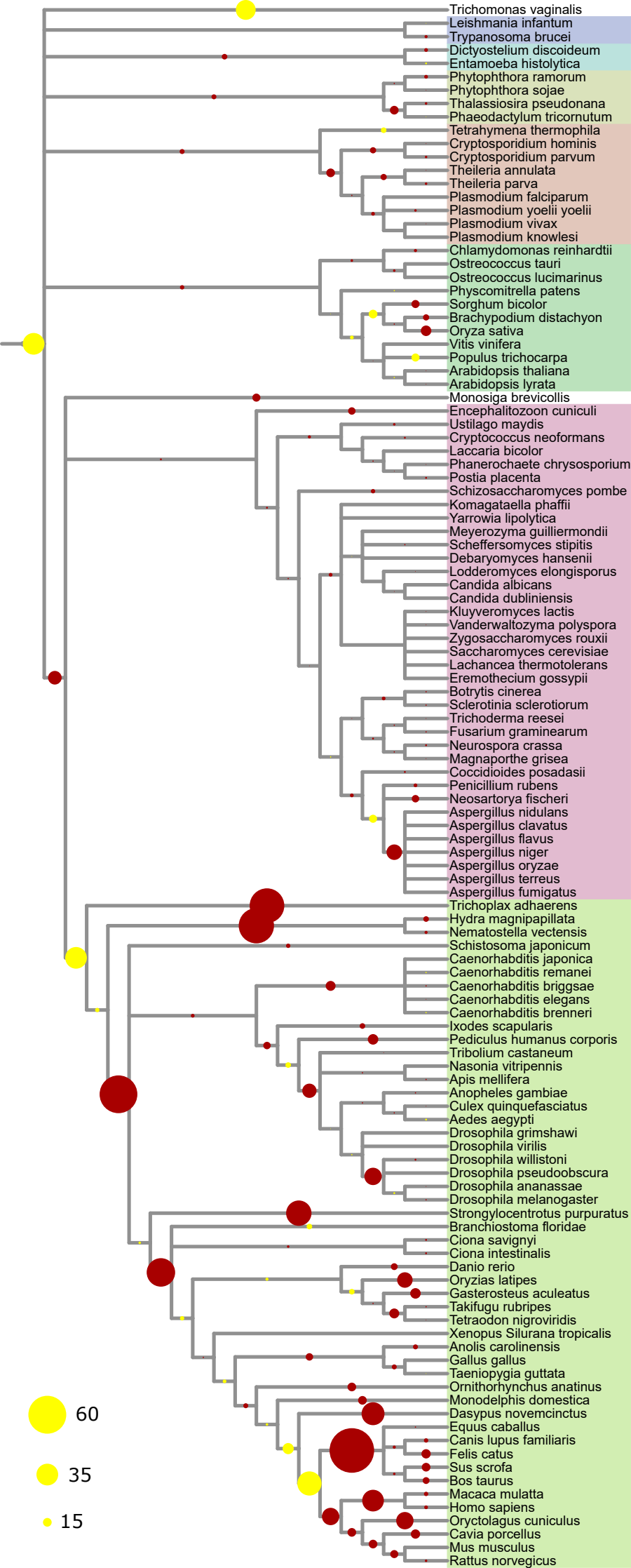

Supplement: Additional file 3: Figure S3. — Tree reconciliation of the DHHC ML tree (without rearrangement). Yellow circles indicate inferred increases in copy numbers of the DHHC family, red circles indicate inferred decreases in copy numbers of the DHHC family. The tree topology is extracted from NCBI taxonomy. (PDF 49 kb) [file 12862_2017_932_MOESM3_ESM.pdf]

**A**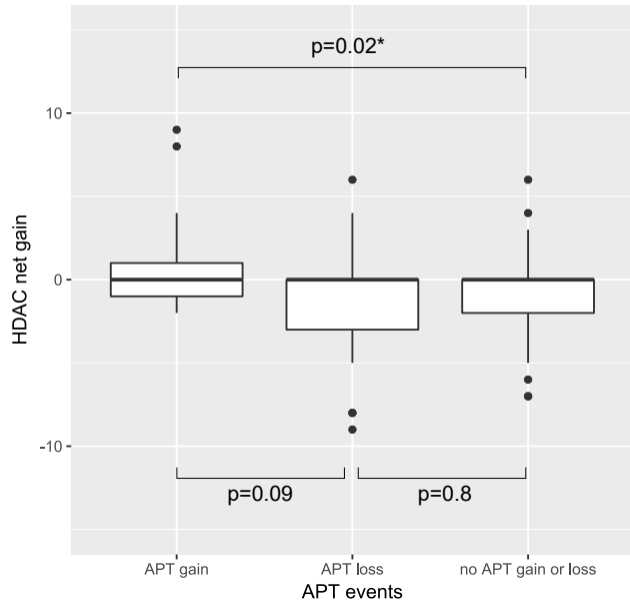**B**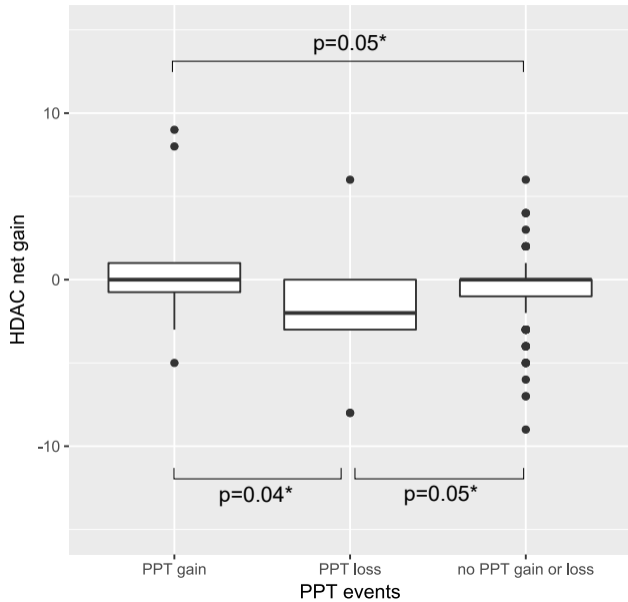

Supplement: Additional file 4: Figure S4. — Histone deacetylase family as a negative control. A) Differences in net gain in HDAC family per branch between three categories of branches, gain in APT, loss in APT or no change in APT family B) Differences in net gain in HDAC family per branch between three categories of branches, gain in PPT, loss in PPT or no change in PPT family (PDF 41 kb) [file 12862_2017_932_MOESM4_ESM.pdf]
